# The channel structure of trithallium penta­anti­m­on­ate(V), Tl_3_Sb_5_O_14_

**DOI:** 10.1107/S2056989022002869

**Published:** 2022-03-17

**Authors:** Paul Sicher, Berthold Stöger

**Affiliations:** a X-Ray Center, TU Wien, Getreidemarkt 9, 1060 Vienna, Austria

**Keywords:** crystal structure, channel structure, anti­monate(V)

## Abstract

Single crystals of Tl_3_Sb_5_O_14_ were grown by solid-state reaction in a corundum crucible under air (1273 K, 12 h). The structure was determined by single-crystal X-ray diffraction. It is isotypic to the K_3_Sb_5_O_14_, Rb_3_Sb_5_O_14_ and Cs_3_Sb_5_O_14_ analogues with ortho­rhom­bic *Pbam* symmetry and cell parameters *a* = 24.2899 (9) Å, *b* = 7.1931 (3) Å, *c* = 7.4182 (3) Å. The Sb atoms form irregular [SbO_6_] octa­hedra, which are linked *via* edges and corners into a triperiodic network. The Tl^+^ ions are located in distinct channels of the network extending along [010] and [001].

## Abstract 2

The channel structure of trithallium penta­anti­monate(V) Tl_3_Sb_5_O_14_ is characterized and compared to the K, Rb and Cs analogues.

## Chemical context

During an extensive study of *M*[SbF_6_] compounds (*M* = Li, NH_4_, Na, Tl), precursors in the form of *M*SbO_3_ were synthesized. Whereas the chosen conditions (1273 K, 12 h) yielded the expected product for LiSbO_3_ and NaSbO_3_, the Tl-poor title compound Tl_3_Sb_5_O_14_ was inadvertently obtained in the case of Tl. TlSbO_3_ was later successfully synthesized at 1073 K. In fact, prior syntheses of TlSbO_3_ were performed at even lower temperatures (Bouchama & Tournoux, 1975 ▸).

The analogues K_3_Sb_5_O_14_ (Hong, 1974 ▸), Rb_3_Sb_5_O_14_ and Cs_3_Sb_5_O_14_ (Hirschle *et al.*, 2001 ▸) have been synthesized at 1373 K using more involved routes. The first structural characterization of K_3_Sb_5_O_14_ was published by Aurivillius (1966 ▸). However, the author gives an incorrect Sohncke space-group symmetry of type *Pba*2, which was later corrected to *Pbam* by (Hong, 1974 ▸).

Hong (1974 ▸) noted unusual enlargement of the atomic displacement parameters (ADP) of K in K_3_Sb_5_O_14_, which are located in distinct channels, suggesting ion conductivity. In fact, the author could partially substitute K for Rb, Ag and Tl in the respective nitrate salt melts. Accordingly, it is expected that the hitherto structurally uncharacterized Ag_3_Sb_5_O_14_ likewise exists. In contrast, substitution with the smaller Na^+^ ion in an NaNO_3_ melt led to a collapse of the structure and formation of the Na-poor Na_2_Sb_4_O_11_. The instability of *M*_3_Sb_5_O_14_ with small ions might explain the successful syntheses of *M*SbO_3_ (*M* = Li, Na) at 1273 K.

## Structural commentary

Tl_3_Sb_5_O_14_ crystallizes in the space group *Pbam* and is isotypic to *M*_3_Sb_5_O_14_ (*M* = K, Rb, Cs). Two different settings of the *Pbam* space group were used to describe the structures: *a* > *b* by Hong (1974 ▸) and *a* < *b* by Hirschle *et al.* (2001 ▸). These are equivalent descriptions, because the (**a**′, **b**′, **c**′) = (**b**, −**a**, **c**) operation is an element of the affine normalizer of the *Pbam* space group. Herein we use the original setting and atom labeling of Hong (1974 ▸).

**Figure 1:**
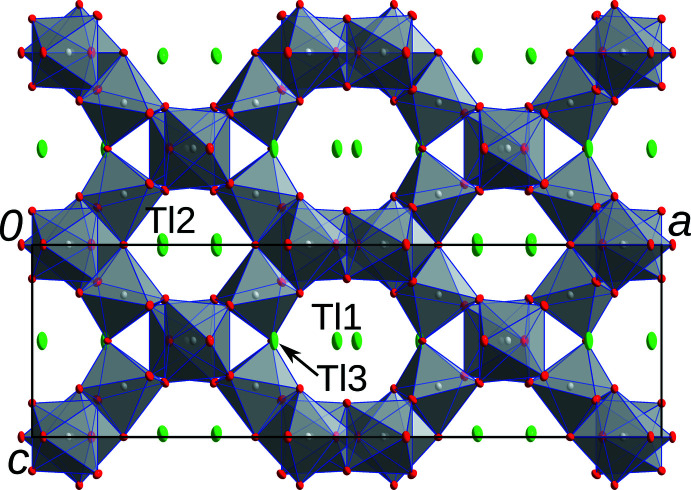
Tl_3_Sb_5_O_14_ viewed down [010], Tl (green), Sb (gray) and O (red) atoms are represented by ellipsoids drawn at the 50% probability level.

**Figure 2:**
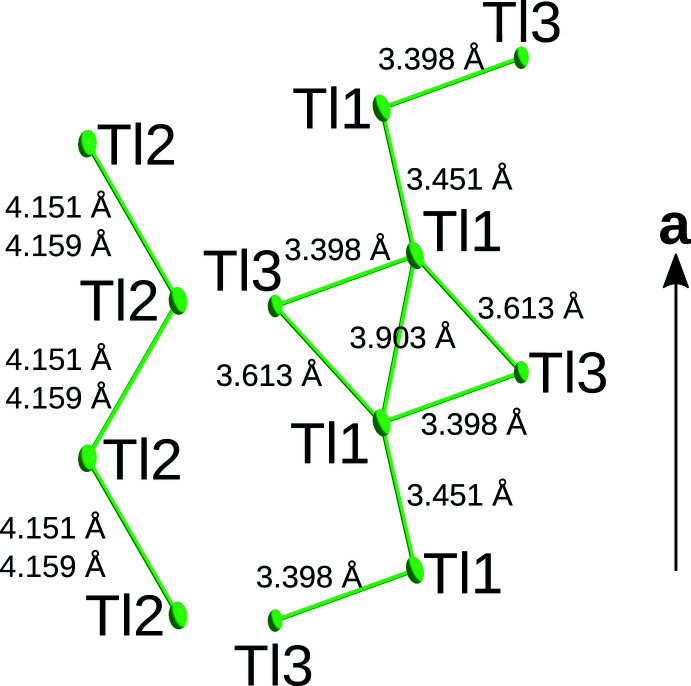
Tl atoms in Tl_3_Sb_5_O_14_ viewed down [001] with inter­atomic distances. For Tl2⋯Tl2 contacts, two inter­atomic distances are given since the Tl2 atom was refined as disordered about the reflection plane parallel to (001).

**Figure 3:**
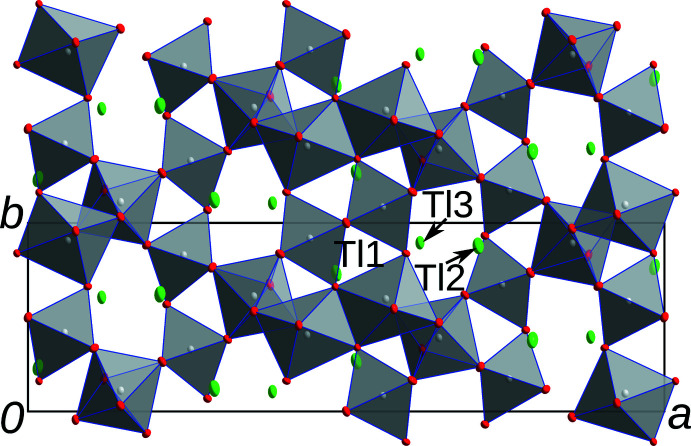
Tl_3_Sb_5_O_14_ viewed down [001]. Atom color codes as in Fig. 1 ▸.

In structures of the *M*_3_Sb_5_O_14_ type, the monovalent metal atoms *M* are located in channels of a triperiodic network formed by [SbO_6_] octa­hedra. There are two distinct channels parallel to [010], both with 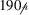 _y_ *b*2_1_ *m* symmetry (Fig. 1 ▸). In one channel, the *M*1 atoms are located in zigzag chains and bridged by the *M*3 atoms, which are located at the boundary of the channels (Fig. 2 ▸). In the second channel, the *M*2 atoms are likewise arranged in the form of zigzag lines (Fig. 2 ▸). All of the *M* atoms are located on or very close to the reflection plane of the channels. Additionally, channels with a smaller diameter extend in the [001] direction (Fig. 3 ▸). For K_3_Sb_5_O_14_, Hong (1974 ▸) reports excessive enlargement of the ADPs of the K1 and K2 atoms in the [010] and [001] directions of the channels, with the ‘thermal motions’ in these directions being ‘eight times bigger’ than in the [100] direction. The Tl1 and Tl2 atoms in the title compound show a much milder enlargement of the ADPs. The ratio of the mean-square displacement of the longest and shortest principal axes of the ADP tensor is 3.2 for Tl1 and 2.9 for Tl2. Note that the value for Tl2 is not directly comparable, since it was refined as disordered about the reflection plane. However, even when placing the atom on the reflection plane, the ratio increases to only 3.2. From these values, it appears that Tl_3_Sb_5_O_14_ is not a prime candidate for ion conductivity, at least at the measurement temperature of 100 K. For Rb_3_Sb_5_O_14_ and Cs_3_Sb_5_O_14_, similarly mild enlargement of the ADPs has been reported (Hirschle *et al.*, 2001 ▸). In contrast to the Tl_3_Sb_5_O_14_ title compound, these were derived from data collected at room temperature.

All Sb atoms are coordinated by six O atoms forming highly irregular [SbO_6_] octa­hedra (Table 1 ▸) with O—Sb—O *cis* angles ranging from 73.37 (17) to 103.83 (13)° and *trans* angles up to 150.66 (16)°. As noted by Hirschle *et al.* (2001 ▸), the framework can be described as being composed of four distinct parts: two infinite octa­hedra chains and two edge-connected pairs of octa­hedra. In general, these elements are connected *via* corners but there is an additional connection between a pair and a chain *via* an edge.

A qu­anti­tative comparison of Tl_3_Sb_5_O_14_ and the alkali-metal analogues *M*_3_Sb_5_O_14_ (*M* = K, Rb, Cs) was performed using the *COMPSTRU* (de la Flor *et al.*, 2016 ▸) module of the Bilbao Crystallographic Server (Aroyo *et al.*, 2006 ▸). The Tl2 atom was moved onto the reflection plane to make the sets of Wyckoff positions compatible. The degree of lattice distortion with respect to the Tl compound is *S* = 0.0042 (*M* = K), *S* = 0.0048 (*M* = Rb) and *S* = 0.0262 (*M* = Cs). This shows that the K, Rb and Tl compounds feature very similar cell parameters, with the volume increasing slightly according to K > Rb > Tl (Table 2 ▸). In contrast, the lattice of Cs_3_Sb_5_O_14_ features a pronounced distortion with a *ca* 11% larger unit-cell volume. The enlargement affects foremost the *a* and *b* lattice parameters, whereas *c* is smaller than for the Tl compound. We therefore presume that the unit-cell volume for the *M* = K, Rb, Tl compounds is mostly determined by the triperiodic anti­monate network, which cannot contract any further. The minimum size of the channels may explain the collapse of the structure when attempting to replace K by Na, as reported by Hong (1974 ▸).

The degree of similarity likewise shows a close relationship of the *M* = K (Δ = 0.022) and *M* = Rb (Δ = 0.035) compounds with Tl_3_Sb_5_O_14_, whereas the atomic positions in Cs_3_Sb_5_O_14_ differ distinctly (Δ = 0.178). In particular, the positions of the O atoms that coordinate to the Tl2 atoms feature a strong deviation (*d*_max_ = 0.6356 Å for the O4 atom) showing a distinct distortion of the [SbO_6_] octa­hedra around the respective channels. Thus, it appears that the Tl2 channels are responsible for the distinct enlargement of the unit cell of Cs_3_Sb_5_O_14_.

## Synthesis and crystallization

A mixture of 0.682 g TlNO_3_ and 0.373 g Sb_2_O_3_ (which makes for an approximate molar ratio of 1:1 for Tl:Sb) was heated in a corundum crucible at 1273 K for 12 h in air. From the reaction, a dark-orange powder was obtained. The single crystals formed as rectangular-prismatic plates. Crystals were isolated under a polarizing microscope and cut to an appropriate size for single crystal diffraction of a highly absorbing crystal.

## Refinement

Crystal data, data collection and structure refinement are summarized in Table 3 ▸. A starting model was generated using the coordinates of K_3_Sb_5_O_14_ (Hong, 1974 ▸). Owing to distinct peaks in the difference-Fourier map, the Tl2 atom was removed from the reflection plane and refined as disordered. Even though the refined distance of the atom from the reflection plane is minute, the residuals improved significantly {*R*[*I* > 2σ(*I*)] from 0.028 to 0.023}, which might be in part due to the increased number of anisotropic displacement parameters.

## Supplementary Material

Crystal structure: contains datablock(s) I, global. DOI: 10.1107/S2056989022002869/pk2663sup1.cif

Structure factors: contains datablock(s) I. DOI: 10.1107/S2056989022002869/pk2663Isup2.hkl

CCDC reference: 2158509

Additional supporting information:  crystallographic information; 3D view; checkCIF report

## Figures and Tables

**Table 1:** Selected geometric parameters (Å, °)

Tl1—Tl3^i^	3.3972 (4)	Sb2—O10	1.919 (3)
Tl1—Tl1^ii^	3.4507 (7)	Sb2—O2^iv^	1.983 (4)
Tl1—Tl3^iii^	3.6130 (4)	Sb2—O2^vii^	2.140 (4)
Tl3—Tl1^iv^	3.3972 (4)	Sb2—O4^vii^	2.215 (4)
Tl3—Tl1^v^	3.6129 (4)	Sb3—O5^iii^	1.952 (4)
Tl1—O3	2.565 (4)	Sb3—O5	1.979 (4)
Tl2—O6	2.775 (4)	Sb3—O9^ix^	1.998 (3)
Tl3—O5	2.495 (4)	Sb3—O9	1.998 (3)
Sb1—O8^vi^	1.925 (3)	Sb3—O7^ix^	2.002 (3)
Sb1—O8	1.925 (3)	Sb3—O7	2.002 (3)
Sb1—O6^vii^	1.971 (4)	Sb4—O3	1.9233 (15)
Sb1—O1^viii^	1.996 (2)	Sb4—O7	1.936 (3)
Sb1—O1	1.996 (2)	Sb4—O9^x^	1.954 (3)
Sb1—O2	2.081 (4)	Sb4—O8	1.975 (3)
Sb2—O6	1.911 (4)	Sb4—O4	2.0284 (11)
Sb2—O10^vi^	1.919 (3)	Sb4—O10^x^	2.041 (3)
			
O8^vi^—Sb1—O8	96.70 (16)	O5^iii^—Sb3—O5	171.04 (9)
O8^vi^—Sb1—O6^vii^	90.34 (11)	O5^iii^—Sb3—O9^ix^	99.04 (11)
O8—Sb1—O6^vii^	90.34 (11)	O5—Sb3—O9^ix^	87.50 (11)
O8^vi^—Sb1—O1^viii^	90.74 (11)	O5^iii^—Sb3—O9	99.03 (11)
O8—Sb1—O1^viii^	171.91 (11)	O5—Sb3—O9	87.50 (11)
O6^vii^—Sb1—O1^viii^	92.82 (8)	O9^ix^—Sb3—O9	85.66 (16)
O8^vi^—Sb1—O1	171.91 (11)	O5^iii^—Sb3—O7^ix^	87.14 (11)
O8—Sb1—O1	90.74 (11)	O5—Sb3—O7^ix^	87.54 (11)
O6^vii^—Sb1—O1	92.82 (8)	O9^ix^—Sb3—O7^ix^	83.44 (11)
O1^viii^—Sb1—O1	81.67 (15)	O9—Sb3—O7^ix^	168.21 (11)
O8^vi^—Sb1—O2	90.17 (11)	O5^iii^—Sb3—O7	87.14 (11)
O8—Sb1—O2	90.17 (11)	O5—Sb3—O7	87.54 (11)
O6^vii^—Sb1—O2	179.23 (15)	O9^ix^—Sb3—O7	168.21 (11)
O1^viii^—Sb1—O2	86.60 (8)	O9—Sb3—O7	83.44 (11)
O1—Sb1—O2	86.60 (8)	O7^ix^—Sb3—O7	107.02 (16)
O6—Sb2—O10^vi^	96.40 (9)	O3—Sb4—O7	93.31 (15)
O6—Sb2—O10	96.40 (9)	O3—Sb4—O9^x^	99.82 (14)
O10^vi^—Sb2—O10	150.66 (16)	O7—Sb4—O9^x^	92.53 (12)
O6—Sb2—O2^iv^	100.16 (16)	O3—Sb4—O8	83.01 (13)
O10^vi^—Sb2—O2^iv^	101.78 (8)	O7—Sb4—O8	88.19 (12)
O10—Sb2—O2^iv^	101.78 (8)	O9^x^—Sb4—O8	177.03 (11)
O6—Sb2—O2^vii^	173.53 (15)	O3—Sb4—O4	160.89 (15)
O10^vi^—Sb2—O2^vii^	85.12 (9)	O7—Sb4—O4	103.83 (13)
O10—Sb2—O2^vii^	85.12 (9)	O9^x^—Sb4—O4	87.96 (13)
O2^iv^—Sb2—O2^vii^	73.37 (17)	O8—Sb4—O4	89.07 (13)
O6—Sb2—O4^vii^	90.22 (16)	O3—Sb4—O10^x^	84.03 (14)
O10^vi^—Sb2—O4^vii^	76.83 (8)	O7—Sb4—O10^x^	177.09 (11)
O10—Sb2—O4^vii^	76.83 (8)	O9^x^—Sb4—O10^x^	89.09 (11)
O2^iv^—Sb2—O4^vii^	169.63 (15)	O8—Sb4—O10^x^	90.31 (11)
O2^vii^—Sb2—O4^vii^	96.25 (14)	O4—Sb4—O10^x^	78.63 (13)

Symmetry codes: (i) 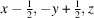 ; (ii) 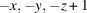 ; (iii) 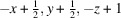 ; (iv) 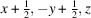 ; (v) 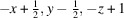 ; (vi) 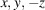 ; (vii) 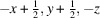 ; (viii) 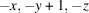 ; (ix) 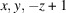 ; (x) 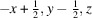 .

**Table 2:** Comparison of unit-cell parameters (Å, Å^3^) of the *M*3Sb_5_O_14_ structures The setting of the *M* = Rb and *M* = Cs compounds was adjusted to the setting used in this work.

Compound	K_3_Sb_5_O_14_	Rb_3_Sb_5_O_14_	Cs_3_Sb_5_O_14_	Tl_3_Sb_5_O_14_
*a*	24.247 (4)	24.478 (2)	26.251 (5)	24.2899 (9)
*b*	7.157 (2)	7.1881 (9)	7.4337 (13)	7.1931 (3)
*c*	7.334 (2)	7.331 (2)	7.396 (3)	7.4182 (3)
*V*	1272.7 (3)	1289.8 (4)	1443.3 (7)	1296.11 (9)

**Table 3:** Experimental details

Crystal data	
Chemical formula	Tl_3_Sb_5_O_14_
*M*_r_	1445.86
Crystal system, space group	Orthorhombic, *P* *b* *a* *m*
Temperature (K)	250
*a*, *b*, *c* (Å)	24.2899 (9), 7.1931 (3), 7.4182 (3)
*V* (Å^3^)	1296.11 (9)
*Z*	4
Radiation type	Mo *K*α
μ (mm^−1^)	47.48
Crystal size (mm)	0.11 × 0.06 × 0.02

Data collection	
Diffractometer	Bruker Kappa APEXII CCD
Absorption correction	Multi-scan (*SADABS*; Bruker, 2021 ▸)
*T*_min_, *T*_max_	0.010, 0.058
No. of measured, independent and observed [*I* > 2σ(*I*)] reflections	27499, 3084, 2850
*R*_int_	0.051
(sin θ/λ)_max_ (Å^−1^)	0.812

Refinement	
*R*[*F* ^2^ > 2σ(*F* ^2^)], *wR*(*F* ^2^), *S*	0.023, 0.055, 1.07
No. of reflections	3084
No. of parameters	121
Δρ_max_, Δρ_min_ (e Å^−3^)	2.55, −1.52

Computer programs: *APEX3* and *SAINT-Plus* (Bruker, 2021 ▸), *SHELXL2014/7* (Sheldrick, 2015 ▸), *DIAMOND* (Putz & Brandenburg, 2021 ▸) and *publCIF* (Westrip, 2010 ▸).

## supplementary crystallographic information

### Crystal data

**Table:**

Tl_3_Sb_5_O_14_	*D*_x_ = 7.410 Mg m^−^^3^
*M**_r_* = 1445.86	Mo *K*α radiation, λ = 0.71073 Å
Orthorhombic, *P**b**a**m*	Cell parameters from 9928 reflections
*a* = 24.2899 (9) Å	θ = 2.8–35.3°
*b* = 7.1931 (3) Å	µ = 47.48 mm^−^^1^
*c* = 7.4182 (3) Å	*T* = 250 K
*V* = 1296.11 (9) Å^3^	Plate, colourless
*Z* = 4	0.11 × 0.06 × 0.02 mm
*F*(000) = 2440	

### Data collection

**Table:**

Bruker Kappa APEXII CCD diffractometer	2850 reflections with *I* > 2σ(*I*)
Graphite monochromator	*R*_int_ = 0.051
ω– and φ–scans	θ_max_ = 35.3°, θ_min_ = 3.0°
Absorption correction: multi-scan (SADABS; Bruker, 2021)	*h* = −39→39
*T*_min_ = 0.010, *T*_max_ = 0.058	*k* = −11→11
27499 measured reflections	*l* = −12→12
3084 independent reflections	

### Refinement

**Table:**

Refinement on *F*^2^	Primary atom site location: isomorphous structure methods
Least-squares matrix: full	*w* = 1/[σ^2^(*F*_o_^2^) + (0.0199*P*)^2^ + 6.584*P*] where *P* = (*F*_o_^2^ + 2*F*_c_^2^)/3
*R*[*F*^2^ > 2σ(*F*^2^)] = 0.023	(Δ/σ)_max_ = 0.001
*wR*(*F*^2^) = 0.055	Δρ_max_ = 2.55 e Å^−^^3^
*S* = 1.07	Δρ_min_ = −1.52 e Å^−^^3^
3084 reflections	Extinction correction: SHELXL-2014/7 (Sheldrick 2015), Fc^*^=kFc[1+0.001xFc^2^λ^3^/sin(2θ)]^-1/4^
121 parameters	Extinction coefficient: 0.00075 (4)
0 restraints	

### Special details

**Table:**

Geometry. All esds (except the esd in the dihedral angle between two l.s. planes) are estimated using the full covariance matrix. The cell esds are taken into account individually in the estimation of esds in distances, angles and torsion angles; correlations between esds in cell parameters are only used when they are defined by crystal symmetry. An approximate (isotropic) treatment of cell esds is used for estimating esds involving l.s. planes.

### Fractional atomic coordinates and isotropic or equivalent isotropic displacement parameters (Å^2^)

**Table:**

	*x*	*y*	*z*	*U*_iso_*/*U*_eq_	Occ. (<1)
Tl1	0.01569 (2)	0.23393 (5)	0.5000	0.03732 (8)	
Tl2	0.29264 (2)	0.12150 (6)	−0.0170 (5)	0.0353 (4)	0.5
Tl3	0.38418 (2)	0.10536 (4)	0.5000	0.03177 (7)	
Sb1	0.05715 (2)	0.41738 (4)	0.0000	0.00993 (6)	
Sb2	0.43805 (2)	0.40456 (4)	0.0000	0.01042 (6)	
Sb3	0.25558 (2)	0.32863 (4)	0.5000	0.00998 (6)	
Sb4	0.14535 (2)	0.11009 (3)	0.26233 (3)	0.01011 (5)	
O1	0.0000	0.5000	0.1759 (5)	0.0131 (6)	
O2	0.01735 (15)	0.1611 (5)	0.0000	0.0130 (6)	
O3	0.11974 (15)	0.1728 (6)	0.5000	0.0139 (6)	
O4	0.14514 (15)	0.0305 (5)	0.0000	0.0124 (6)	
O5	0.28203 (16)	0.0685 (5)	0.5000	0.0146 (6)	
O6	0.40613 (16)	0.1618 (5)	0.0000	0.0146 (6)	
O7	0.21049 (11)	0.2637 (4)	0.2830 (4)	0.0144 (5)	
O8	0.10390 (11)	0.3355 (4)	0.1939 (4)	0.0138 (4)	
O9	0.31369 (11)	0.3832 (4)	0.3169 (4)	0.0136 (4)	
O10	0.42520 (10)	0.4563 (4)	0.2502 (3)	0.0132 (4)	

### Atomic displacement parameters (Å^2^)

**Table:**

	*U*^11^	*U*^22^	*U*^33^	*U*^12^	*U*^13^	*U*^23^
Tl1	0.01779 (11)	0.04162 (17)	0.05255 (18)	0.00612 (10)	0.000	0.000
Tl2	0.01922 (12)	0.04252 (19)	0.0442 (12)	−0.00204 (11)	−0.0027 (2)	0.0106 (5)
Tl3	0.01232 (10)	0.02797 (13)	0.05504 (18)	0.00031 (8)	0.000	0.000
Sb1	0.00754 (12)	0.01037 (13)	0.01187 (12)	0.00045 (9)	0.000	0.000
Sb2	0.00826 (12)	0.01106 (13)	0.01193 (12)	0.00000 (9)	0.000	0.000
Sb3	0.00756 (11)	0.01032 (13)	0.01205 (12)	−0.00028 (9)	0.000	0.000
Sb4	0.00803 (9)	0.01136 (10)	0.01094 (9)	0.00039 (6)	−0.00072 (6)	−0.00010 (7)
O1	0.0106 (14)	0.0163 (16)	0.0123 (13)	0.0028 (12)	0.000	0.000
O2	0.0080 (14)	0.0098 (15)	0.0214 (16)	−0.0005 (11)	0.000	0.000
O3	0.0107 (15)	0.0223 (18)	0.0086 (13)	0.0045 (13)	0.000	0.000
O4	0.0119 (14)	0.0150 (16)	0.0101 (13)	−0.0016 (12)	0.000	0.000
O5	0.0114 (15)	0.0106 (15)	0.0217 (16)	−0.0008 (12)	0.000	0.000
O6	0.0113 (15)	0.0087 (15)	0.0238 (17)	−0.0011 (12)	0.000	0.000
O7	0.0123 (11)	0.0159 (12)	0.0150 (10)	−0.0035 (9)	−0.0031 (9)	0.0011 (9)
O8	0.0142 (11)	0.0128 (11)	0.0144 (10)	0.0034 (9)	−0.0038 (9)	−0.0013 (9)
O9	0.0119 (10)	0.0125 (11)	0.0165 (10)	−0.0029 (8)	0.0023 (9)	−0.0003 (9)
O10	0.0093 (10)	0.0161 (11)	0.0141 (10)	0.0003 (8)	0.0005 (8)	0.0001 (9)

### Geometric parameters (Å, º)

**Table:**

Tl1—Tl3^i^	3.3972 (4)	Sb2—Sb2^x^	3.3079 (6)
Tl1—Tl1^ii^	3.4507 (7)	Sb3—O5^iii^	1.952 (4)
Tl1—Tl3^iii^	3.6130 (4)	Sb3—O5	1.979 (4)
Tl2—Tl2^iv^	0.252 (7)	Sb3—O9^xi^	1.998 (3)
Tl3—Tl1^v^	3.3972 (4)	Sb3—O9	1.998 (3)
Tl3—Tl1^vi^	3.6129 (4)	Sb3—O7^xi^	2.002 (3)
Tl1—O3	2.565 (4)	Sb3—O7	2.002 (3)
Tl2—O6	2.775 (4)	Sb4—O3	1.9233 (15)
Tl3—O5	2.495 (4)	Sb4—O7	1.936 (3)
Tl3—Sb3	3.5123 (4)	Sb4—O9^xii^	1.954 (3)
Sb1—O8^iv^	1.925 (3)	Sb4—O8	1.975 (3)
Sb1—O8	1.925 (3)	Sb4—O4	2.0284 (11)
Sb1—O6^vii^	1.971 (4)	Sb4—O10^xii^	2.041 (3)
Sb1—O1^viii^	1.996 (2)	Sb4—Sb2^xiii^	3.1743 (3)
Sb1—O1	1.996 (2)	O1—Sb1^viii^	1.996 (2)
Sb1—O2	2.081 (4)	O2—Sb2^i^	1.983 (4)
Sb1—Sb1^viii^	3.0199 (6)	O2—Sb2^xiii^	2.140 (4)
Sb2—O6	1.911 (4)	O3—Sb4^xi^	1.9233 (15)
Sb2—O10^iv^	1.919 (3)	O4—Sb4^iv^	2.0283 (11)
Sb2—O10	1.919 (3)	O4—Sb2^xiii^	2.215 (4)
Sb2—O2^v^	1.983 (4)	O5—Sb3^vi^	1.952 (4)
Sb2—O2^vii^	2.140 (4)	O6—Sb1^xiii^	1.971 (4)
Sb2—O4^vii^	2.215 (4)	O6—Tl2^iv^	2.775 (4)
Sb2—Sb4^vii^	3.1742 (3)	O9—Sb4^ix^	1.954 (3)
Sb2—Sb4^ix^	3.1742 (3)	O10—Sb4^ix^	2.042 (3)
			
O3—Tl1—Tl3^i^	169.98 (10)	Sb4^vii^—Sb2—Sb2^x^	112.786 (12)
O3—Tl1—Tl1^ii^	92.89 (10)	Sb4^ix^—Sb2—Sb2^x^	112.786 (12)
Tl3^i^—Tl1—Tl1^ii^	97.130 (13)	O5^iii^—Sb3—O5	171.04 (9)
O3—Tl1—Tl3^iii^	57.56 (10)	O5^iii^—Sb3—O9^xi^	99.04 (11)
Tl3^i^—Tl1—Tl3^iii^	112.419 (11)	O5—Sb3—O9^xi^	87.50 (11)
Tl1^ii^—Tl1—Tl3^iii^	150.451 (14)	O5^iii^—Sb3—O9	99.03 (11)
Tl2^iv^—Tl2—O6	87.40 (7)	O5—Sb3—O9	87.50 (11)
O5—Tl3—Tl1^v^	166.21 (9)	O9^xi^—Sb3—O9	85.66 (16)
O5—Tl3—Sb3	33.32 (9)	O5^iii^—Sb3—O7^xi^	87.14 (11)
Tl1^v^—Tl3—Sb3	132.896 (12)	O5—Sb3—O7^xi^	87.54 (11)
O5—Tl3—Tl1^vi^	126.21 (9)	O9^xi^—Sb3—O7^xi^	83.44 (11)
Tl1^v^—Tl3—Tl1^vi^	67.581 (11)	O9—Sb3—O7^xi^	168.21 (11)
Sb3—Tl3—Tl1^vi^	159.523 (11)	O5^iii^—Sb3—O7	87.14 (11)
O8^iv^—Sb1—O8	96.70 (16)	O5—Sb3—O7	87.54 (11)
O8^iv^—Sb1—O6^vii^	90.34 (11)	O9^xi^—Sb3—O7	168.21 (11)
O8—Sb1—O6^vii^	90.34 (11)	O9—Sb3—O7	83.44 (11)
O8^iv^—Sb1—O1^viii^	90.74 (11)	O7^xi^—Sb3—O7	107.02 (16)
O8—Sb1—O1^viii^	171.91 (11)	O5^iii^—Sb3—Tl3	145.11 (12)
O6^vii^—Sb1—O1^viii^	92.82 (8)	O5—Sb3—Tl3	43.84 (11)
O8^iv^—Sb1—O1	171.91 (11)	O9^xi^—Sb3—Tl3	57.42 (8)
O8—Sb1—O1	90.74 (11)	O9—Sb3—Tl3	57.42 (8)
O6^vii^—Sb1—O1	92.82 (8)	O7^xi^—Sb3—Tl3	112.32 (8)
O1^viii^—Sb1—O1	81.67 (15)	O7—Sb3—Tl3	112.32 (8)
O8^iv^—Sb1—O2	90.17 (11)	O3—Sb4—O7	93.31 (15)
O8—Sb1—O2	90.17 (11)	O3—Sb4—O9^xii^	99.82 (14)
O6^vii^—Sb1—O2	179.23 (15)	O7—Sb4—O9^xii^	92.53 (12)
O1^viii^—Sb1—O2	86.60 (8)	O3—Sb4—O8	83.01 (13)
O1—Sb1—O2	86.60 (8)	O7—Sb4—O8	88.19 (12)
O8^iv^—Sb1—Sb1^viii^	131.51 (8)	O9^xii^—Sb4—O8	177.03 (11)
O8—Sb1—Sb1^viii^	131.51 (8)	O3—Sb4—O4	160.89 (15)
O6^vii^—Sb1—Sb1^viii^	93.72 (11)	O7—Sb4—O4	103.83 (13)
O1^viii^—Sb1—Sb1^viii^	40.84 (8)	O9^xii^—Sb4—O4	87.96 (13)
O1—Sb1—Sb1^viii^	40.84 (8)	O8—Sb4—O4	89.07 (13)
O2—Sb1—Sb1^viii^	85.51 (10)	O3—Sb4—O10^xii^	84.03 (14)
O6—Sb2—O10^iv^	96.40 (9)	O7—Sb4—O10^xii^	177.09 (11)
O6—Sb2—O10	96.40 (9)	O9^xii^—Sb4—O10^xii^	89.09 (11)
O10^iv^—Sb2—O10	150.66 (16)	O8—Sb4—O10^xii^	90.31 (11)
O6—Sb2—O2^v^	100.16 (16)	O4—Sb4—O10^xii^	78.63 (13)
O10^iv^—Sb2—O2^v^	101.78 (8)	O3—Sb4—Sb2^xiii^	117.70 (12)
O10—Sb2—O2^v^	101.78 (8)	O7—Sb4—Sb2^xiii^	146.72 (8)
O6—Sb2—O2^vii^	173.53 (15)	O9^xii^—Sb4—Sb2^xiii^	93.61 (8)
O10^iv^—Sb2—O2^vii^	85.12 (9)	O8—Sb4—Sb2^xiii^	84.23 (8)
O10—Sb2—O2^vii^	85.12 (9)	O4—Sb4—Sb2^xiii^	43.86 (10)
O2^v^—Sb2—O2^vii^	73.37 (17)	O10^xii^—Sb4—Sb2^xiii^	35.42 (7)
O6—Sb2—O4^vii^	90.22 (16)	Sb1^viii^—O1—Sb1	98.33 (15)
O10^iv^—Sb2—O4^vii^	76.83 (8)	Sb2^i^—O2—Sb1	131.46 (19)
O10—Sb2—O4^vii^	76.83 (8)	Sb2^i^—O2—Sb2^xiii^	106.63 (17)
O2^v^—Sb2—O4^vii^	169.63 (15)	Sb1—O2—Sb2^xiii^	121.92 (17)
O2^vii^—Sb2—O4^vii^	96.25 (14)	Sb4—O3—Sb4^xi^	132.9 (2)
O6—Sb2—Sb4^vii^	99.60 (9)	Sb4—O3—Tl1	111.03 (11)
O10^iv^—Sb2—Sb4^vii^	38.07 (8)	Sb4^xi^—O3—Tl1	111.03 (11)
O10—Sb2—Sb4^vii^	113.51 (8)	Sb4^iv^—O4—Sb4	147.2 (2)
O2^v^—Sb2—Sb4^vii^	136.95 (5)	Sb4^iv^—O4—Sb2^xiii^	96.76 (11)
O2^vii^—Sb2—Sb4^vii^	85.49 (8)	Sb4—O4—Sb2^xiii^	96.76 (11)
O4^vii^—Sb2—Sb4^vii^	39.39 (3)	Sb3^vi^—O5—Sb3	133.2 (2)
O6—Sb2—Sb4^ix^	99.60 (9)	Sb3^vi^—O5—Tl3	124.01 (18)
O10^iv^—Sb2—Sb4^ix^	113.51 (8)	Sb3—O5—Tl3	102.84 (16)
O10—Sb2—Sb4^ix^	38.07 (8)	Sb2—O6—Sb1^xiii^	129.2 (2)
O2^v^—Sb2—Sb4^ix^	136.95 (5)	Sb2—O6—Tl2	119.90 (17)
O2^vii^—Sb2—Sb4^ix^	85.49 (8)	Sb1^xiii^—O6—Tl2	110.88 (16)
O4^vii^—Sb2—Sb4^ix^	39.39 (3)	Sb2—O6—Tl2^iv^	119.90 (17)
Sb4^vii^—Sb2—Sb4^ix^	75.620 (11)	Sb1^xiii^—O6—Tl2^iv^	110.88 (16)
O6—Sb2—Sb2^x^	138.46 (12)	Tl2—O6—Tl2^iv^	5.21 (15)
O10^iv^—Sb2—Sb2^x^	93.86 (8)	Sb4—O7—Sb3	130.09 (14)
O10—Sb2—Sb2^x^	93.86 (8)	Sb1—O8—Sb4	138.08 (15)
O2^v^—Sb2—Sb2^x^	38.31 (11)	Sb4^ix^—O9—Sb3	131.67 (14)
O2^vii^—Sb2—Sb2^x^	35.07 (10)	Sb2—O10—Sb4^ix^	106.51 (12)
O4^vii^—Sb2—Sb2^x^	131.32 (10)		

Symmetry codes: (i) *x*−1/2, −*y*+1/2, *z*; (ii) −*x*, −*y*, −*z*+1; (iii) −*x*+1/2, *y*+1/2, −*z*+1; (iv) *x*, *y*, −*z*; (v) *x*+1/2, −*y*+1/2, *z*; (vi) −*x*+1/2, *y*−1/2, −*z*+1; (vii) −*x*+1/2, *y*+1/2, −*z*; (viii) −*x*, −*y*+1, −*z*; (ix) −*x*+1/2, *y*+1/2, *z*; (x) −*x*+1, −*y*+1, −*z*; (xi) *x*, *y*, −*z*+1; (xii) −*x*+1/2, *y*−1/2, *z*; (xiii) −*x*+1/2, *y*−1/2, −*z*.
